# Thermo-Statistical Effects of Inclusions on Vesicles: Division into Multispheres and Polyhedral Deformation

**DOI:** 10.3390/membranes12060608

**Published:** 2022-06-11

**Authors:** Yuno Natsume

**Affiliations:** 1 Schoolteacher Training Course/Natural Sciences, Cooperative Faculty of Education, Utsunomiya University, Mine-machi 350, Utsunomiya 321-8505, Japan; natsumey@cc.utsunomiya-u.ac.jp; 2 Institute for Promotion of Research Center for Bioscience Research and Education, Utsunomiya University, Mine-machi 350, Utsunomiya 321-8505, Japan

**Keywords:** vesicle, division, beads, membrane elastic energy, depletion interaction, osmotic pressure, Alder transition

## Abstract

The construction of simple cellular models has attracted much attention as a way to explore the origin of life or elucidate the mechanisms of cell division. In the absence of complex regulatory systems, some bacteria spontaneously divide through thermostatistically elucidated mechanisms, and incorporating these simple physical principles could help to construct primitive or artificial cells. Because thermodynamic interactions play an essential role in such mechanisms, this review discusses the thermodynamic aspects of spontaneous division models of vesicles that contain a high density of inclusions, with their membrane serving as a boundary. Vesicles with highly dense inclusions are deformed according to the volume-to-area ratio. The phase separation of beads at specific intermediate volume fractions and the associated polyhedral deformation of the membrane are considered in relation to the Alder transition. Current advances in the development of a membrane-growth vesicular model are summarized. The thermostatistical understanding of these mechanisms could become a cornerstone for the construction of vesicular models that display spontaneous cell division.

## 1. Introduction

The cell—the smallest unit of life—is full of organelles [1,2,3,4] that are involved in various complex dynamics such as cell division [5] and even in prokaryotic cells, cell division is handled by specialized proteins such as FtsZ [6]. In contrast, L-form bacteria, which have membranes but no cell walls or cytoskeleton, divide in a disorganized manner [7] and their division is often regarded as a model for early life. The principles of simple modes of cell division have been extensively investigated in fields related to the origin of life and artificial cells. Primitive cells might have been compartmentalized by deformable boundaries without definite cell walls or cytoskeletons [8,9,10]. As primitive cell models, lipid membrane compartments such as micelles [10,11], emulsions [10], and vesicles [12] are often used and generally have a small membrane elastic modulus. In systems where rigid inclusions such as coiled polymers or hard spherical particles are confined within deformable compartments, the elastic energy of the microemulsion membrane together with the translational entropy of the internal materials results in membrane deformation [13]. In the exploration of a vesicle system, Dinsmore et al. found that when nanometer-scale beads of two different sizes are highly confined, the membrane curvature changes [14]. These data suggest that membrane deformation is caused by depletion interaction, a local osmotic effect between inclusions [14,15]. Natsume et al. used centrifugation to swell thin phospholipid films with bead dispersion or oil-in-water emulsion to create vesicles that encapsulated a high density of beads in a confined area [16,17,18] and they observed dynamic vesicle deformations such as division into multispheres and angular shapes [16,19,20]. These data suggest that even if the internal beads are all of one type, the depletion interaction between them and the vesicle membrane can cause vesicle deformation [16,19]. A theoretical study that used the Monte Carlo simulations of the interactions between encapsulated beads described the relationship between the various deformations of the boundary membrane and bead localization [21].

One can imagine that the entropic or thermostatistical effects of inclusions have also contributed to the deformation and division of primitive cells. The division of L-form bacteria is due to membrane growth and subsequent instability, which suggests it is caused by a thermostatistical effect [7]. Vesicles encapsulating polymers also divide, presumably because of a decrease in osmotic pressure with a consequent increase in the entropy of the internal polymer [22] and such a vesicle division system without a specific mechanism could be a model to understand the division of L-form bacteria [23,24] and the self-reproduction of primitive cells [25]. Even in division models with functional proteins such as FtsZ, it is essential to reduce the osmotic pressure of the inclusions [26,27]. Therefore, considering the mechanical effects of the inclusions is important [28,29,30].

This review summarizes the spontaneous cell division models based on vesicles with confined materials from the viewpoint of thermostatistical mechanics, focusing on the author’s work as a member of the Imai group [16] and the Toyota group [19,20], as well as some unpublished work. The following topics are discussed: the total energy of vesicles that contain inclusions (Section 2); the cooperative action between the elastic energy of the membrane and the free energy of inclusions (Section 3); the division of vesicles containing highly confined microspheres (Section 4); and the polyhedral deformation of vesicles containing microspheres at specific volume fractions (Section 5). Section 6 is a summary, and Section 7 describes vesicle systems that mimic L-form bacteria as a direction for future research.

## 2. Total Energy of Vesicles

In classical studies, empty vesicles were deformed by external factors such as osmotic pressure [31,32], changes in temperature [33,34], and the fusion of multiple vesicles [35], and the elastic energy of the membrane has only been considered [36,37]. In systems with inclusions, the total energy needs to be estimated and would be the sum of the elastic energy of the compartment and the free energy of the inclusions.

The following parameters are used to describe the elastic energy of the vesicular membrane: its elastic modulus, its area, and the vesicle curvature. The shape of the vesicle is affected by the membrane elastic energy and the area-to-volume ratio (*ξ*, dimensionless) as indices [37]: *ξ*= *R_s_/R_v_* − 1; *R_s_* = ${\left(A/4\pi \right)}^{\frac{1}{2}}$, where *R_s_* is the radius of a hypothetical sphere whose area is *A*; *R_v_* = ${\left(3V/4\pi \right)}^{\frac{1}{3}}$, where *R_v_* is the radius of a hypothetical sphere whose volume is *V*. For a perfect sphere, *ξ* = 0; for other shapes, *ξ* is positive.

The free energy of confined particles is closely related to their translational motion. It depends on the free volume in which they can freely move [38,39]. Free energy also depends on the number of inclusions and the temperature, which are the same before and after the division. When the free volume of a particle inside a vesicle is taken into account, the center of mass of the particle cannot be closer to the inner surface of the vesicle membrane than to its radius [14,40]. This inaccessible region is called the depletion region, and its volume is denoted as *V_dep_*. Accordingly, the free volume of the inclusions is the volume of the vesicle minus that of the depletion region (*V_dep_*).

## 3. Cooperativeness of Elastic Energy of Membrane and Free Energy of Inclusions

For the free energy of inclusions to be dominant in vesicle deformation, the number and volume of inclusions must be large. Two feasible strategies allow inclusions to contribute to deformation: (i) increasing the inclusion’s size and (ii) increasing the number of inclusions. It is challenging to encapsulate a large number of rigid micrometer-sized spheres in vesicles because each vesicle has a finite volume, but it has been achieved in the author’s experiments [16,17,18,19,20].

To increase the inclusion size, Natsume et al. encapsulated a few hundred to a few thousand one-micrometer polystyrene beads into vesicles of ten to several tens of micrometers in diameter [16,19]. Polymers could be regarded as hard spheres with a diameter of several to several tens of nanometers, which is two to three orders of magnitude smaller than the diameter of these polystyrene beads; however, the above number of beads per vesicle is just enough to consider the thermostatistical effects.

To increase the number of inclusions, Terasawa et al. encapsulated 5 w% polyethylene glycol (PEG) with a sufficient gyration radius into vesicles [22]; in the PEG-encapsulating vesicles that mimicked the cell wall and intracellular biopolymers, they observed cell division-like deformation. These vesicles spontaneously deformed after fusion and eventually had a dumbbell shape consisting of two spherical vesicles. Terasawa et al. also assessed whether the depletion interaction considerably contributed to the deformation. The free energy change of the inner particles by vesicle deformation (|Δ*E_dep_*|) was calculated as ΔΠΔ*V_dep_*, in which ΔΠ was the osmotic pressure of the encapsulated PEG. Under their division-like deformation condition of the vesicles and the confined PEG, the relationship between |Δ*E_dep_*| and the elastic energy change of the vesicle membrane |Δ*E_bend_*| holds as |Δ*E_dep_*| >> |Δ*E_bend_*|, and the deformation reduces PEG free energy.

## 4. Division of Vesicles with Densely Encapsulated Microspheres

The previous section briefly mentioned two strategies to construct spontaneous cell division models and described one of those strategies, as realized by Terasawa et al.’s experimental system [22]. This section describes the other strategy in which vesicles with a higher volume fraction (approximately 50 vol%) are more efficiently constructed by encapsulating one-micrometer polystyrene beads into the model.

As mentioned in Section 2, the depletion region depends on the size of the inclusions. Micrometer-sized or larger beads can be observed under a microscope, and the dynamics of the membrane and beads can be simultaneously observed in real time.

### 4.1. Change in Vesicle Volume

**Figure 1:**
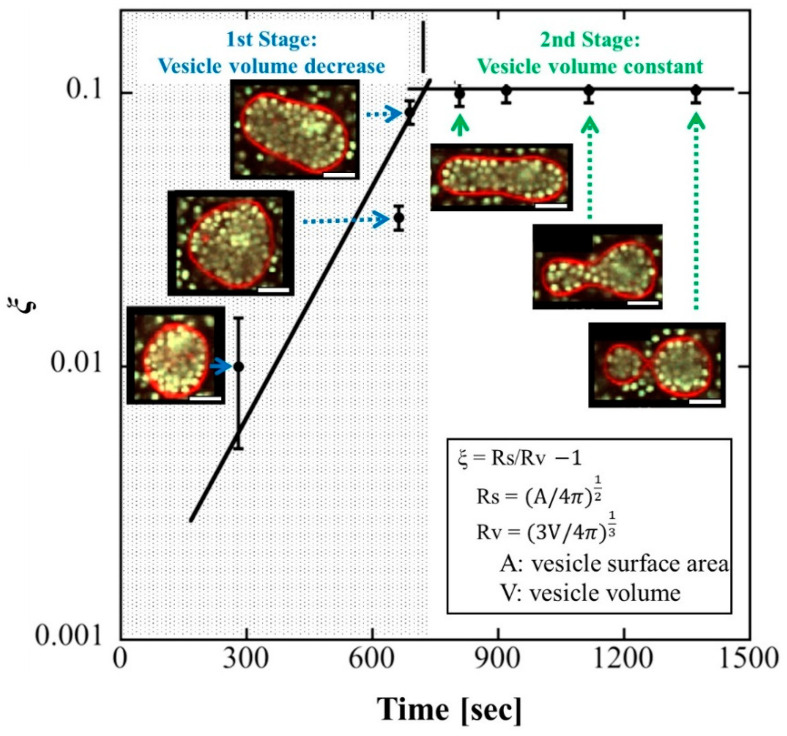
Shape deformation pathway of a vesicle containing densely encapsulated polystyrene beads. Scale bars, 5 μm. A perfect sphere has *ξ =* 0. Adding a hypertonic solution caused a decrease in the vesicle volume and an increase in *ξ* in the first stage (<700 s). In the second stage, where *ξ* was almost constant, the vesicle was deformed into two connected spheres. This figure is revised from Figure 2 in [16].

Natsume et al. created vesicles with densely packed beads by using centrifugation when swelling a phospholipid film with a dilute bead dispersion [16]. Vesicle deformation via osmotic pressure was induced by adding a hypertonic solution, and for over 10 min (the first stage in Figure 1), the vesicles had characteristic features (the first stage in Figure 1) such as prolate, oblate, and stomatocyte shapes, as observed in empty vesicles. This stage was followed by the sequential formation of multispherical vesicles, with no other stable vesicle shapes (the second stage in Figure 1). The area-to-volume ratio *ξ* was used to analyze the morphological changes (Figure 1). The vesicles were spherical and stable when they were prepared (*ξ =* 0). The addition of a hypertonic solution caused water to flow out of the vesicles, resulting in a decrease in the vesicle volume while the membrane area remained constant (*ξ* increased). As a result of the osmotic pressure, the vesicle was stretched along its axis and became tubular. It reached the limit of *ξ* increase at approximately 700 s, and the tubular vesicle deformed into two spheres connected by a narrow neck at approximately 1400 s. The vesicle volume remained almost constant between 700 s and 1400 s.

The shape of two spheres has higher elastic energy than that of a tubule. This suggests that elastic energy was not responsible for the observed deformation. The free volume of the encapsulated beads was estimated as *V_free_* = *V* − *V_dep_*, where *V* is the total volume of the vesicle and *V_dep_* is the volume of the depletion region, which covers the inner surface of the vesicle membrane at a width equivalent to a bead’s radius. At the same volume, the two spheres had a larger *V_free_* than that of the tubule. Therefore, to maximize the translational entropy of the confined beads, the vesicles were deformed from a tubule into two connected spheres.

### 4.2. Division Corresponding to the Area-to-Volume Ratio

**Figure 2:**
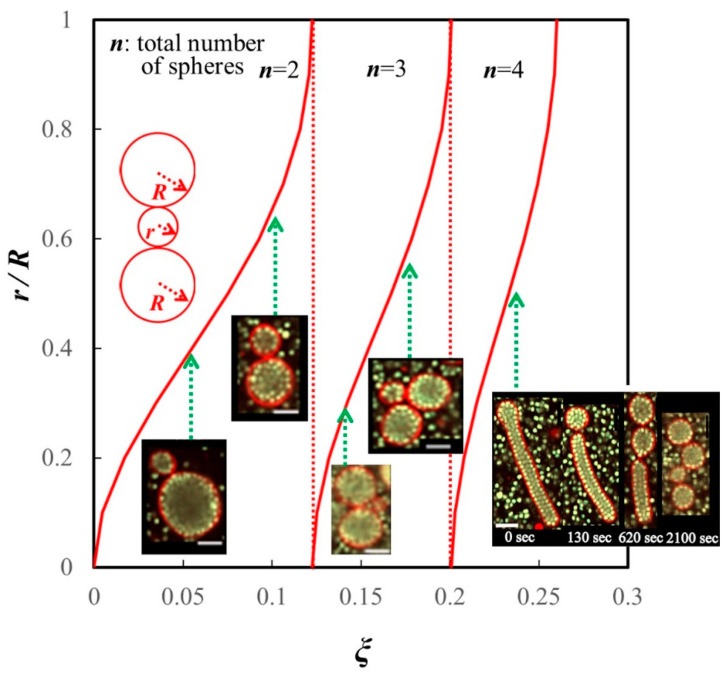
Geometrical relationship between the ratio of radii *r/R* and *ξ* for the divided vesicles that encapsulated polystyrene beads. *R*, the radius of large spheres when *n* = 2, 3, and 4; *r*, radius of a single small sphere. Calculated results (red curves) were in good agreement with the experimental data (micrographs). The right micrograph shows a typical shape deformation pathway from a tubular vesicle to multispheres when *n* = 4. Scale bars, 5 μm. At *n* ≥ 3, vesicles divided into one small sphere and other (*n* − 1) spheres that all had the same radius. This figure is a revision of Figures 6 and 7 in [16].

Natsume et al. also described the shape deformation of long tubular vesicles encapsulating dense beads with *ξ* > 0.12. Such vesicles divided into multispherical vesicles— consisting of three or four spheres from each tubule [16]. Tubular vesicles sequentially divided into spheres from one or both ends over several minutes (Figure 2). Given that the multispherical vesicles consisted of *n* – 1 spheres, each with a radius *R*, and one small sphere with radius *r* (*r* < *R*), the ratio of *r* to *R*, as well as *n*, is uniquely determined by *ξ* (Figure 2). There was good agreement between the vesicle deformation and geometrical prediction using *ξ* [16].

Similar to the estimate for the vesicles that divided into two spheres (see Section 4.1), vesicles that divided into three or four spheres had a larger free volume than that of a tubular vesicle of the same volume [16].

### 4.3. Restriction of Water Outflow by Inclusions

Natsume et al.’s work on the deformation of tubular vesicles raised some questions, namely: how is the radius is determined and why are sequentially formed spheres not of the same size when the entropy of beads is maximized? These questions can be explained as follows. In the process of vesicle deformation, spheres with the same *R* as the curvature of the tubule end are initially created from each end. Spheres of this size continue to be created until the ratio of area to volume in the tubule eventually needs to be adjusted by the creation of a smaller sphere. This system does not require an external control for division, and *ξ* can determine the number and size of spheres to be produced. Spontaneously forming a curvature that gives some patterned multispheres was discussed in relation to osmotic pressure [41].

To comprehend the importance of *ξ* in the vesicle division, the relationship between *ξ* and the osmotic effect of inclusions should be understood. In the classical method of inducing vesicle deformation by applying osmotic pressure to drain water from the interior, the value of *ξ* is determined by the reduction in vesicle volume. When osmotic pressure is applied to a vesicle containing polymers or beads, the decrease in volume resulting from water outflow inevitably increases the polymer or bead concentration or volume fraction. However, only limited research has been conducted on the relationship between ξ and the osmotic effect of inclusions. When Fujiwara et al. performed experiments in which cell extracts were confined to vesicles and concentrated by osmotic pressure, they found that the water outflow was restricted as the density approached a cell-like level (approximately 30 vol%) [42,43]. No further increase in density occurred, even when osmotic pressure was further increased [42]. In Natsume et al.’s experimental system, vesicles with various ξ values were divided (Figure 2). Immediately after preparation, almost all vesicles were spherical (*ξ* = 0), and *ξ* increased with the outflow of water. Our findings that vesicles were densely packed at the time of division and that the decrease in volume almost stopped before and after division suggest that the volume fraction of the inclusions limits water outflow.

Thus, the entropy effect essentially contributes to the encapsulated polystyrene beads system with a volume fraction of approximately 50 vol%. This system, which is based on the area-to-volume ratio of the vesicle, displayed the characteristic behavior of regular divisions into multispheres, and in the system, the osmotic effect of the encapsulated beads seems to contribute to the change in vesicle volume.

## 5. Polyhedral Deformation of Vesicles Encapsulating Microspheres at Specific Volume Fractions

This type of deformation accompanies the characteristic arrangement of internal beads at volume fractions of approximately 15 vol%.

### 5.1. Phase Separation from the Perspective of Alder Transition

Volume fraction is an important parameter in bead dispersion. At low values, beads are randomly dispersed, but as the volume fraction increases, beads form two phases, namely ordered and disordered, which coexist in a crystal–fluid state. In a finite volume, the volume of the partially crystallized fraction is lower than that of the randomly close-packed fraction because the randomly dispersed beads have higher entropy [44]. The loss of entropy due to crystallization in one part of the system is compensated by an increase in entropy as the density decreases in the remaining fluid part. This phase separation has been discussed from the perspective of the Alder transition [44,45,46]. In polystyrene beads confined in vesicles, this phase separation occurs as the volume fraction of the beads is reduced [19,20]. This behavior affects the deformation of the membrane.

### 5.2. Coexistence of Ordered and Disordered Phases of Microspheres in Vesicles

**Figure 3:**
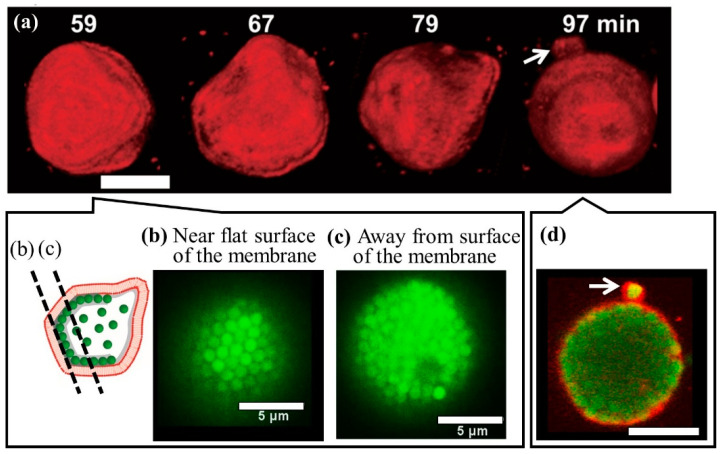
Temporal transition of an asymmetrical polyhedral vesicle at 59–97 min after the addition of hypertonic solution. (**a**) The deformation pathway of a vesicle with encapsulated polystyrene beads at 13 vol%. Scale bar, 5 μm. (**b**,**c**) Behavior of the encapsulated polystyrene beads near or away from the flat surface of the vesicle membrane at 59 min; although the beads in (**b**) are organized in a crystallization pattern, no orderly bead array could be found in (**c**). (**d**) A tubular structure (white arrow) protruding from the polyhedral vesicle at 97 min. Moderate volume fractions of polystyrene beads play an essential role in this characteristic polyhedral deformation. The figure is modified from Figures 3 and 4 in [19].

To prepare vesicles containing polystyrene beads with volume fractions ranging from 0 to 45 vol%, Natsume et al. adopted the water-in-oil emulsion transfer method using centrifugation [17,18]. A hypertonic aqueous solution of d-glucose was added to induce the polyhedral deformation of the vesicles; a typical example of the deformation is shown in Figure 3. Polyhedral deformation was mostly observed in vesicles with a diameter of approximately 20 μm and a volume fraction of approximately 13 vol%. The angles corresponding to the polyhedral were obtained at the corners of the flat surfaces. This was followed by the formation of a prominent protrusion at the angular antipodal point, as seen in Figure 3d [19]. This characteristic configuration was explained by the coexistence of crystallized and disordered phases of the beads in the vesicles. Several triangular faces of bead crystals were formed on the vesicle membrane. The characteristic patterns of colloidal particles observed on such faces correspond to close-packed structures and suggest an orderly array of particles [20].

It has been well documented that phase separation owing to Alder transition is unique to finite systems [47], and its existence cannot be theoretically proven for infinite systems. Vesicles encapsulating beads with a specific volume fraction are finite systems. The present system is a typical example of a multiphase system caused by Alder transition.

## 6. Conclusions

This review discussed the use of vesicles that encapsulate beads or polymers as a division model based on thermostatistical mechanics [16,19,22]. Vesicles with highly dense inclusions divide into several small spherical vesicles [16,22]. Given that the depletion region exists on the inner surface of the vesicle membrane and has a width equivalent to the radius of the beads or rounded polymers, divisions reduce the osmotic pressure and increase the entropy of the inclusions by decreasing the depletion region. In a vesicle encapsulating beads at moderate volume fractions [19], beads adjacent to the vesicle inner membrane transiently become regularly aligned [20]. During division, planes form along the vesicle membrane in which beads are arranged in an orderly pattern, probably because the thermostatistical effect of increasing the free volume of disordered beads increases the entropy of the internal beads as a whole. In both cases, the focus has been on spontaneous deformation at a constant membrane area-to-volume ratio. Vesicles with a continuously increasing membrane area would be a better model to mimic cell division and are considered in the next section.

## 7. Future Directions

In the systems presented herein, bulky excess membranes were produced to induce vesicle deformation by fusing vesicles or draining water through changes in osmotic pressure. Because of these ways to give an area-to-volume ratio *ξ*, the membrane surface area of the vesicle was taken as a sporadic or unchanging value determined at the time of preparation. However, the membrane surface area continuously increases during cell division. In eukaryotes, the total membrane area increases as phospholipids are synthesized in the endoplasmic reticulum and are distributed throughout the cell. Subsequently, a contractile ring composed of motor proteins causes the plasma membrane to be progressively constricted. This leads to the completion of cytokinesis [5]. Membrane growth comes up as an essential problem to take note of in the study of cell division models [48,49].

Systems in which the membrane area increases have been reported [50,51,52,53]. Walde et al. showed that when insoluble oleic anhydride is present in a 100 nm oleic acid vesicle dispersion, the vesicles catalyze the hydrolysis of the anhydride to produce more oleic acid molecules [54]. Newly produced oleic acid is incorporated into the existing vesicles, increasing their size to approximately 500 nm. A similar increase in membrane volume was observed when fatty acid micelles were introduced into a solution of fatty acid vesicles [55].

The author’s group is also constructing a system that increases the number of membrane molecules in the vesicle to mimic L-form bacteria. The addition of sodium oleate to micrometer-size phospholipid vesicles increased the membrane area by up to 50% in almost all vesicles [56]. These findings indicate that even if a vesicle does not have a biosynthetic pathway for amphiphilic molecules, the membrane area can be spontaneously increased by the insertion of heterogeneous amphiphilic molecules into the vesicular membrane. Various deformation paths were induced by adding sodium oleate to vesicles containing the PEG of different molecular weight, but not to empty vesicles. However, unlike the systems of Terasawa et al. [22] or Natsume et al. [16], no division into three or more spheres was observed, even when the area-to-volume ratio (ξ) is sufficiently large. Therefore, deformation paths depend on whether the membrane surface area continuously increases or remains constant. Dividing vesicle systems with membrane enlargement have been constructed to make the vesicles bear greater resemblance to living cells. To realize primitive cellular life models based on vesicles, it is essential for a vesicle with highly dense inclusions to acquire such features as autocatalysis [57] or self-replication [58,59].

Membrane deformation induced by inclusions has been studied in various systems [60] such as deformation by active inclusions [61,62] or bundles of filaments [63,64]. In these studies, membrane deformations are attributed to the properties of the inclusions. From the viewpoint of the thermostatistical effect, it is possible to deform a membrane even if the inclusions do not have individual properties, as described in this review—namely, their entropic effects play an essential role [26,28,29,30]. The combination of membrane deformation caused by a continuous increase in surface area and the entropic effect of inclusions could lead to deformation patterns that settle into a specific stable state, resulting in behaviors that are characteristic when specialized proteins are absent. Regardless of the molecular species, thermostatistical studies are likely to be essential in realizing these behaviors.
